# Genetic Variability of *Aspergillus flavus* Isolates from a Mississippi Corn Field

**DOI:** 10.1155/2014/356059

**Published:** 2014-11-12

**Authors:** Cesar D. Solorzano, Hamed K. Abbas, Robert M. Zablotowicz, Perng-Kuang Chang, Walker A. Jones

**Affiliations:** ^1^Biological Control of Pests Research Unit, United States Department of Agriculture, Agricultural Research Service, Stoneville, MS 38776, USA; ^2^Crop Production Systems Research Unit, United States Department of Agriculture, Agricultural Research Service, Stoneville, MS 38776, USA; ^3^Food and Feed Safety Research Unit, United States Department of Agriculture, Agricultural Research Service, New Orleans, LA 70124, USA

## Abstract

A nontoxigenic *Aspergillus flavus* strain, K49, is currently being tested as a biological control agent in corn fields in the Mississippi Delta. However, little is known about the overall genetic diversity of *A. flavus* from year to year in corn fields and specifically in Mississippi. Our objective was to assess the genetic variability of *A. flavus* isolates from different seasons, inoculum sources, and years, from a no-till corn field. Of the 175 *A. flavus* isolates examined, 74 and 97 had the typical *norB-cypA* type I (1.5 kb) and type II (1.0 kb) deletion patterns, respectively. Variability in the sequence of the *omtA* gene of the majority of the field isolates (*n* = 118) was compared to strain K49. High levels of haplotypic diversity (24 *omtA* haplotypes; Hd = 0.61 ± 0.04) were found. Among the 24 haplotypes, two were predominant, H1 (*n* = 71), which consists of mostly toxigenic isolates, and H49 (*n* = 18), which consists of mostly atoxigenic isolates including K49. Toxigenic isolates were prevalent (60%) in this natural population. Nonetheless, about 15% of the population likely shared the same ancestral origin with K49. This study provides valuable information on the diversity of *A. flavus*. This knowledge can be further used to develop additional biological control strains.

## 1. Introduction

Corn,* Zea mays* L., is a major agricultural crop in the state of Mississippi and in the U.S., with an estimated acreage of 770,000 and 91,897,000 acres planted in 2011, respectively [1]. One major food safety concern for corn, peanuts, rice, and edible tree nuts is the contamination with toxins produced by* Aspergillus flavus *Link [2].* Aspergillus flavus* is a soil-borne haploid fungus, known to asexually produce conidia and sclerotia and recently reported to have a sexual stage [3, 4]. The fungus and other* Aspergillus* spp. in section* Flavi* can synthesize aflatoxins and cyclopiazonic acid (CPA) [5, 6]. Cyclopiazonic acid is able to inhibit mammalian calcium-ATPase and interfere with Ca^2+^ level in animal systems [7]. Aflatoxins B_1_ (AFB_1_) and B_2_ (AFB_2_) are produced by* A. flavus*, and aflatoxins G_1_ (AFG_1_) and G_2_ (AFG_2_) in addition to the B aflatoxins are produced by* A. parasiticus* [8–10].* A. flavus *isolates with large deletions of the aflatoxin gene cluster and portions of the subtelomeric region generally result in no aflatoxin and/or CPA production [11].

Aflatoxins can be more problematic for corn grain and residues in the southern U.S. than other regions due to subtropical climate and summer precipitation deficit of the former region [12, 13]. In addition, 70% of corn production uses some form of reduced tillage, therefore increasing the corn residues contaminated with* A. flavus* left in the field for the next growing season. The U.S. Food and Drug Administration (FDA) regulatory limit for aflatoxin levels in corn feed used in finishing beef cattle is 300 ng/g and for corn used for human consumption is 20 ng/g [14, 15].

A biological control approach to reduce the impact that toxigenic* A. flavus* strains have on corn production is to use nontoxigenic* A. flavus* strains to displace the toxigenic strains in the field [16]. These nontoxigenic strains include K49 (NRRL 30797) and the commercially available AF36 (NRRL 18543) and afla-guard (NRRL21882) which are approved by U.S. Environmental Protection Agency (EPA) for use in a variety of crops in the U.S. These strains competitively exclude aflatoxin producers by applying them to soil and plant parts during the vegetative growth stages [17]. International efforts in Argentina [18], Nigeria [19], and China [20] are currently underway to identify prospective biocontrol strains.

Aflatoxins in* A. flavus* strains are synthesized by a group of approximately 25 genes that form the aflatoxin gene cluster, a region spanning 65–70 kb [10, 25]. Genetic variability occurs in the aflatoxin gene cluster of* A. flavus* isolates. Partial gene deletions in the* norB-cypA* gene region result in known deletion patterns I and II [26]. The biocontrol strain AF36 contains all genes in the aflatoxin biosynthesis gene cluster, with a 90% homology to that of aflatoxin producing* A. flavus* [27]. The basis for its nontoxigenicity is a single nucleotide mutation that results in a stop codon near the beginning of the coding sequence of* pksA* which encodes a polyketide synthase [28]. Previous studies have confirmed that K49 also contains the whole aflatoxin gene cluster [29]. A recent study reports that the same* pksA* missense mutation occurs in K49 [30].

A variety of techniques have been used to examine population diversity of toxigenic and nontoxigenic strains present in various crops and geographical locations. They include amplified fragment length polymorphism (AFLP) [31], random amplified fragment polymorphism (RAPD) [32, 33], direct sequencing, intersimple sequence repeats (ISSR) [34], and microsatellite markers [20, 35, 36]. Eight deletion patterns in the aflatoxin gene cluster have been reported for a group of 38 nonaflatoxigenic* A. flavus* isolates [37]. In a group of soil field isolates from Mississippi corn fields, 60% of* A. flavus* isolated had the potential to produce aflatoxins and all isolates expressed* aflD*,* aflG*,* aflP*,* aflR*, and* aflS* aflatoxin genes [29]. Recently, the presence of mating type genes has been determined (*MAT1-1* or* MAT1-2*) in* A. flavus* [38]. Moreover, another important target of research is the quantification of* A. flavus* by real-time PCR that targets the* nor-1* gene from the aflatoxin gene cluster [39].

The objective of this research was to assess the temporal genetic variability of* A. flavus* isolates from a no-tilled corn field in Mississippi using two markers,* norB-cypA* and* omtA *genes from the aflatoxin gene cluster. Single nucleotide polymorphism (SNP) in the* omtA* gene has been shown to have a sufficient discriminatory power in typing strains of* A. flavus *and closely related* A. oryzae* [37]. Knowledge of this genetic information will help to better understand the community of isolates in a given corn field with no previous exposure to nontoxigenic strains and to determine if additional local isolates could be developed for biological control programs.

## 2. Materials and Methods

### 2.1. Collection of* Aspergillus flavus* Isolates and Confirmation of Aflatoxin Production

One hundred-seventy five* A. flavus* isolates were obtained from soil, plant parts (e.g. silk, leaves, cobs, seeds, and tassels), and adult beetles collected randomly from a no-till Bt hybrid corn field in Elizabeth, MS, during the growing (May–October) and overwintering (December–April) seasons of 2006–2009. Soil isolates were obtained as described elsewhere by diluting 10 g of soil into 150 mL of a solution of 2 g of agar/L of water [40]. 100 *μ*L of each sample was transferred to modified dichloronitroaniline rose bengal agar (MDRB) with 3% NaCl and the inoculum was distributed uniformly with a sterile spreader and incubated at 37°C for 5 days [41, 42]. Putative* A. flavus* isolates were transferred into potato dextrose agar (PDA) plates amended with 0.3%*β*-cyclodextrin to enhance fluorescence and incubated at 28°C for 5 days in the dark.* Aspergillus flavus* identification was based on morphological characteristics [43]. Isolates were examined for fluorescence under UV light (365 nm) and subjected to biochemical aflatoxin confirmation with the ELISA Veratox test kit [12, 44].

### 2.2. DNA Extraction, PCR Amplification, and Sequencing

A single agar plug containing spores was transferred into a sterile 2 mL centrifuge tube containing 1 mL of 0.05% Triton X-100 solution. The tube was vortexed vigorously and 100 *μ*L of the spore suspension was inoculated into 25 mL of potato dextrose broth (PDB) in a 50 mL plastic tube. Tubes were incubated horizontally for 4-5 days at 30°C at 150 rpm.

After visible fungal growth, mycelia was vacuum-dried. Approximately 100–150 mg of wet mycelia was used for DNA extraction using the ZR Fungal/Bacterial DNA kit (Zymo Research, Orange County, CA). DNA extraction followed the manufacturer's instructions in which cells were lysed by bead beating for 5 min with a Disruptor Genie (Zymo Research, Orange County, CA). Eluted DNA was the template for PCR amplification of partial fragments of* norB-cypA* (300/800 bp) and* omtA *(594 bp) genes of the aflatoxin gene cluster and* ITS* (600 bp) gene regions. Platinum Taq DNA Polymerase High Fidelity (Life Technologies, 5791 Van Allen Way, Carlsbad, CA, 92008) was used for the PCR reaction.

Primer pairs were norB-cypA-F 5′-GTGCCCAGCATCTTGGTCCA-3′, norB-cypA-R 5′-AGGACTTGATGATTCCTCGTC-3′ [27], omtA-F 5′-CAGGATATCATTGTGGACGG-3′, omtA-R 5′-CTCCTCTACCAGTGGCTTCG-3′ [36], ITS1 5′-TCCGTAGGTGAACCTGCGG-3′, and ITS4 5′-TCCTCCGCTTATTGATATGC-3′ [20]. ITS sequences of selected samples were used for species confirmation. PCR was conducted following the profile of 2 min at 95°C, followed by 30 cycles of 94°C for 60 s, 45°C for 60 s, and 72°C for 90 s, with a final extension of 72°C for 5 min. Amplicons were separated in 1.5% agarose gels in TBE and photographed using a BioDoc-It Imaging System (UVP Inc., Upland, CA). PCR products for sequencing were gel extracted using Quantum Prep Freeze 'N Squeeze DNA Gel extraction spin columns (Biorad Laboratories, Hercules, CA) and sequenced at the Genomics and Bioinformatics Research Unit of the USDA-ARS in Stoneville, Mississippi.

### 2.3. Alignment, Population Genetic Analysis, and Population Structure

DNA sequences were aligned with SeqMan Pro in DNASTAR (Madison, WI).* omtA *haplotypes were determined manually. The program DNAsp version 5.10.01 [45] was used to estimate population genetic parameters: haplotype diversity (Hd), nucleotide diversity (*π*), mean number of pair wise nucleotide differences (*K*), theta per site (*θ*_*s*_), and theta per gene (*θ*_*g*_). Levels of gene flow were determined through the effective number of migrants (*N*_*m*_) according to the formula of Nei [24] across years and inoculum source. Parameters that test for population expansion or selection: *D* ^+^ and *F* ^+^ statistics [21], Fu's* F's* statistic, Strobeck *S* statistic [22], and Tajima's* D* [23] statistic, were also calculated with DNAsp.

The program Arlequin version 3.5.1.2 [46] calculated nucleotide differences between years and inoculum sources by estimating *F*_ST_ values and determined genetic structure with an analysis of molecular variance (AMOVA) with the methodology from Excoffier et al. [47]. Phylogenetic relationships among isolates were established using the Maximum Likelihood (ML) analysis method in MEGA version 5 [48].

## 3. Results

### 3.1. Confirmation of Isolates and Deletion Patterns of the* norB-cypA* Gene Region

All 175 isolates were confirmed with morphological characteristics and also by* ITS* sequence data (>99.5% homology) to be* A. flavus*. Conidia of isolates appeared with a characteristic yellowish green to darker green. A total of 105 isolates that were positive for UV fluorescence were also positive for ELISA aflatoxin detection, and 66 were not positive for UV fluorescence and ELISA (Table 1). Amplification of the partial* norB-cypA* gene region also confirmed that isolates were* A. flavus*. From a total of 175 isolates, 171 were amplified for the* norB-cypA* gene region. The norB-cypA F/R primer pair generated a 300 bp and an 800 bp fragment for 74 and 97* A. flavus* isolates that correspond to the 1.5 kb (type I) and a 1.0 kb (type II) deletion patterns, respectively (Table 1).

### 3.2. Genetic Analysis of* omtA* Gene Region

A total of 118* A. flavus* isolates (67% of *n* = 175 isolates) were selected for* omtA* sequence analysis (Table 1). These isolates were selected because their* omtA* gene fragments were clearly amplified as revealed by gel electrophoresis. The subsequent SNP analysis identified 24* omtA *haplotypes. Seventy-one of the 118 isolates belonged to a single haplotype designated as haplotype “1” (H1). This haplotype was observed in 10 of the 12 season/source/year locations. Likewise, haplotype H49, designated “49” because its sequence is exactly the same as K49, was found in 18 isolates in 9 of the 12 season/source/year locations but was not found in plant debris 2006–2008.

Haplotype diversity was high (Hd = 0.61) across sites and ranged from 0.22 to 0.86 (Table 2). The variable number of nucleotide sites (*S*) totaled 68 from the 597 nucleotide sites analyzed. Of those, singleton variable sites were 31 and parsimony informative sites were 37. Nucleotide diversity (*π*) was low, ranging between 0.001 and 0.025 with an average of 0.018. Other nucleotide diversity parameters were *K* = 11.08, *θ*_*s*_ = 0.023, and *θ*_*g*_ = 13.94 for the total population. The hypothesis of neutral mutation was tested with statistical parameters *D* ^+^, *F* ^+^, *Fs*, Strobeck's *S*, and Tajima's *D*. Except for a few parameters, most were not significant at *P* < 0.05, and hence no assumptions on population growth or selection can be made. Plant debris isolates from the overwintering season of 2007 appear to be most genetically distinct when compared to all other populations. *N*_*M*_ in these comparisons was low (≤1.29), which demonstrates low levels of gene flow, and 8 of the 11 *F*_ST_ comparisons of this group of isolates were significantly different from all other populations tested (Table 3).

### 3.3. Population Structure

AMOVA partitioned the variation among years, among populations within years and within populations. Variation was mostly explained by within populations (84.92%) (df = 106, *F*_ST_ = 0.15, *P* < 0.0009). Variation among populations within years accounted for 20.63% of the variation (df = 8, *F*_SC_ = 0.19, *P* < 0.0009) and there was no variation among years (0%) (df = 3, *F*_CT_ = −0.05, *P* < 0.56).

### 3.4. Phylogenetic Analysis

**Figure 1:**
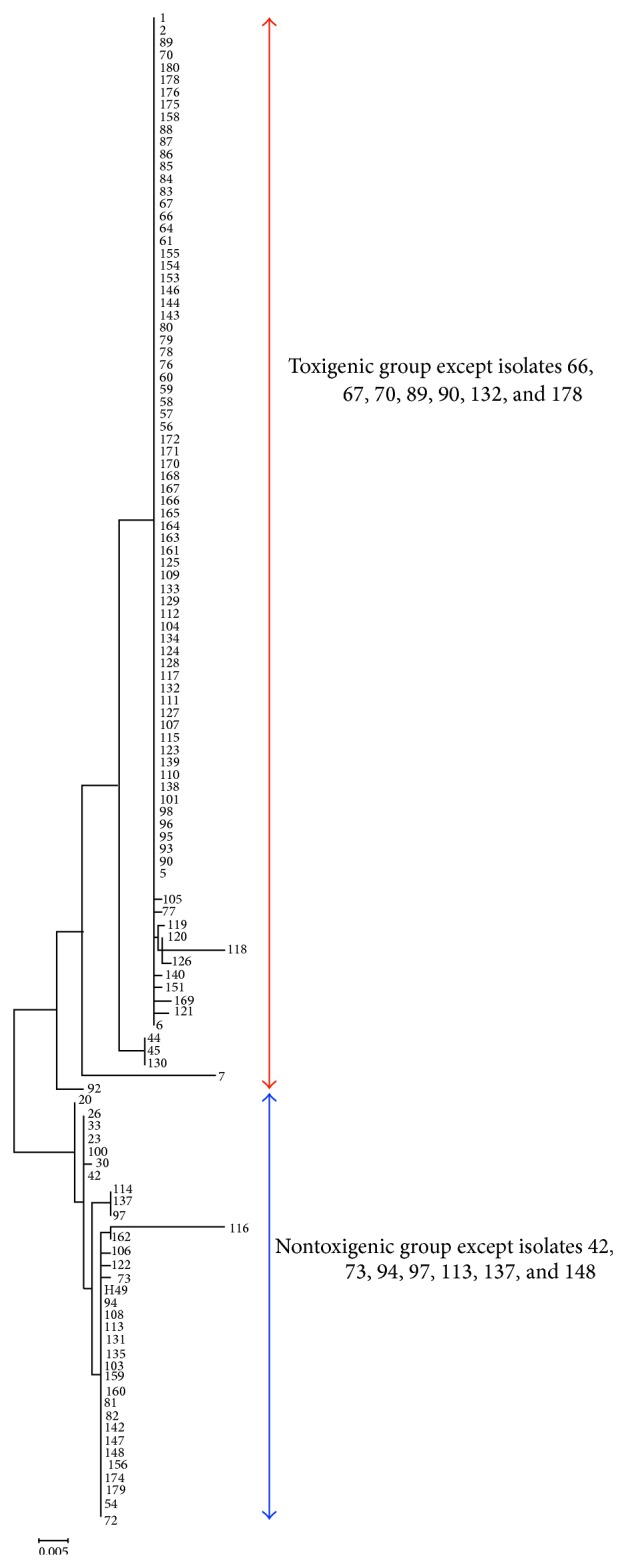
Maximum likelihood analysis of* omtA* gene sequences for 118* A. flavus* collected from a single field in Mississippi 2006–2009. (Scale is 0.005.)

Our ML phylogenetic analysis showed that isolates were not clustered based on the season, inoculum source, or year (Figure 1). For example, isolates 159 to 172 were collected from soil between January and April in 2006 (Table 1), but isolates of 161 and 163–172 are in a clade different from that of isolates 159, 160, and 162. Nonetheless, the 118 isolates formed two major clades based on the aflatoxin producing ability. H1 (isolates 1 to 5) is toxigenic and found to be most distantly related to H49 (H49 to 72). The branch that contains H1, at the upper part of the genealogy tree, includes the group of isolates that were confirmed to be toxigenic. Only 6 isolates within this group were nontoxigenic (isolates 66, 67, 70, 89, 90, 132, and 178) and however closely related to toxigenic isolates by analysis of the* omtA* gene region. Likewise, there were 7 toxigenic isolates (42, 73, 94, 97, 113, 137, and 148) among the 34 isolates that grouped within the nontoxigenic branch where H49 was positioned. These isolates are closely related to H49 but result in production of aflatoxin.

## 4. Discussion

In this study we assessed the population genetic diversity of* A. flavus* across years from a single field in Mississippi and determined how this population of isolates is related to the native Mississippi nontoxigenic isolate K49. H49 was present in 10 of the 12 locations, and therefore these isolates could present a shared origin. The other six isolates that were closely related to H49 were nontoxigenic and they may have mutations or deletions in other genes with the aflatoxin gene cluster that result in nontoxigenicity. We found that a high proportion (60%) of the populations are toxigenic isolates of haplotype H1. These H1 toxigenic isolates are present in all years and inoculum sources. In contrast, Abbas et al. [40] observed a higher percent of toxigenic* A. flavus* isolates in soil than stover and cobs with grain; cobs had the lowest percent of toxigenic* A. flavus*. We speculate that this no-till field with continuous corn production has supported a buildup of a higher number of H1 toxigenic isolates over time probably in part due to their better competitiveness against* A. flavus* isolates of other haplotypes.

*Aspergillus flavus* populations from a corn field community can be diverse due to large amounts of propagule materials [49]. Conidia can be carried through air movement, and sclerotia can remain in the soil for the next growing season. However, we found that, in addition to H1, haplotype H49 is prevalent (15%) in soil and above ground sources but not in plant debris. The biocontrol strain K49 also belongs to the H49 haplotype. Hence, we can assume that H49 isolates from this Mississippi field share an ancestral origin with K49, which was isolated from corn in Mississippi [41]. H49 isolates are present in 10 of the 12 locations examined, which suggests their adaptability and survivability in the field. Other biocontrol candidate strains may be further selected from this particular haplotype. Application of a mixture of highly competitive biocontrol* A. flavus* strains has become a new paradigm for mitigating preharvest aflatoxin problems in some parts of world, especially in Nigeria [Joseph Atehnkeng personal communication, also see http://www.aflasafe.com/ for details].

Our somewhat limited sample size might explain why a large number of haplotypes were found only once as evidence by the high levels of haplotype diversity. The importance and relative frequency of these single-isolate haplotypes cannot be ascertained unless a larger sample size is taken into consideration. Isolates, detected only once, might have an origin beyond this field and could have been carried in by wind or insects, thereby increasing the genetic diversity of this field. Besides H1 and H49, H7 and H8 are the only other haplotypes found in more than one source. H7 was found in the growing season of 2006 in both soil and above ground inoculum, and H8 was found during the overwintering seasons of 2007 and 2008. Given that there was no structure found across years, variation in the populations cannot be explained as a year effect, but within a year, populations are variable according to their source.

Bayman and Cotty [50] found that 66/105 of* A. flavus* soil isolates comprised 13 vegetative compatibility groups in a single Arizona cotton field over a 3-year period and one VCG includes 20% of all isolates from the field. Unlike Gashgari et al. [51], who used RAPD analysis, we are able to differentiate toxigenic from nontoxigenic isolates using both analytical methods of UV detection and ELISA and, to a lesser extent, with the partial* omtA* gene sequence. Others like Midorikawa et al. [33] have been able to characterize* A. flavus* populations based on host plant. In the context of* A. flavus* diversity from a single corn field, the* omtA* marker provided a satisfactory level of resolution. Geiser et al. [52, 53] and Chang et al. [37] included this genetic marker as part of their studies when comparing* A. flavus* and* A. oryzae* isolates. Yin et al. [20] also included this genetic marker in the study that examined nontoxigenic peanut* A. flavus* isolates from a field in China. They found five deletion patterns in 56 toxigenic and nontoxigenic isolates from peanut fields and included 11 genes from the aflatoxin gene cluster in their analysis. The* omtA *and* norB-cypA* gene regions can be more commonly used by researchers, as a way to genetically characterize* A. flavus* isolates. The majority of genes in the aflatoxin gene cluster are highly conserved, as demonstrated by Ehrlich et al. [54] for* A. flavus* and the related aflatoxin producers of* A. nomius* and* A. parasiticus*. The atoxigenic isolates that are clustered with other toxigenic isolates (Figure 1) may have mutations or deletions in genes in the aflatoxin gene cluster, which results in nontoxigenicity [26].

This study provides useful information to the scientific community that studies* A. flavus* populations. The knowledge of* A. flavus *genetic diversity can be used to develop additional biocontrol agents native to the local agricultural areas. Further work will determine if these atoxigenic H1 isolates are genetically similar to other genes within the aflatoxin gene cluster, specially the* pksA* gene that contains the missense mutation that results in nontoxigenicity in the biocontrol strains K49 and AF36.

## Figures and Tables

**Table 1:** *Aspergillus flavus* isolates collected in Elizabeth, MS, from 2006 through 2009, UV fluorescence and ELISA aflatoxin detection (+/−), *norB-cypA* amplicon fragment size, and *omtA* haplotype frequency.

Sources	Time period	AF (+)	AF (−)	*norB-cypA* patterns	*omtA* haplotypes (frequency)
Soil	Jan.–Apr. 2006	140, 161, 163–172	159, 160, 162, 173	4^b^ 12^c^	H1 (10)^d^, H6 (1), H14 (1), H17 (1), H49 (2)
	May–Oct. 2006	93–99	90–92	4^b^ 6^c^	H1 (5), H7 (1), H11 (1), H49 (1)
	Jan.–Apr. 2007	56–58, 60, 76–80, 141, 143–146, 148, 149, 153–155	59, 81, 82, 142, 147, 150–152, 156	7^b^ 21^c^	H1 (15), H16 (1), H19 (1), H49 (6)
	May–Oct. 2007	101, 105, 107, 110, 115, 123, 138, 139	100, 108	2^b^ 8^c^	H1 (7), H8 (1), H18 (1), H49 (1)
	Jan.–Apr. 2008	61, 62, 64, 83–88, 157, 158, 175–177, 180	63, 65–67, 174, 178, 179	6^b^ 14^c^	H1 (15), H49 (2)
	May–Oct. 2008	111, 113, 116, 117, 120, 121, 127, 136	132	3^b^ 6^c^	H1 (4), H5 (1), H15 (1), H21 (1), H49 (1)
	Jan.–Apr. 2009	55, 73	50–54, 68–72, 74, 75, 89	8^b^ 7^c^	H1 (2), H2 (1), H49 (2)

Above ground^a^	May–Oct. 2006	124, 128, 134, 137	102, 106, 114, 122, 131, 135	6^b^ 4^c^	H1 (3), H3 (1), H4 (1), H7 (2), H49 (2)
	May–Oct. 2008	104, 109, 112, 118, 125, 126, 129, 133	103, 119	2^b^ 8^c^	H1 (6), H20 (1), H22 (1), H23 (1), H49 (1)

Plant debris	Jan.–Apr. 2006	1–8	9–14	7^b^ 7^c^	H1 (4), H12 (1)
	Jan.–Apr. 2007	19, 22, 24–26, 28, 29	16–18, 20, 21, 23, 27, 30	15^b^	H8 (2), H9 (1), H10 (1)
	Jan.–Apr. 2008	34, 36–38, 40–45	31–33, 35, 39	11^b^ 4^c^	H8 (2), H13 (2)

Combined		105	66	74^b^, 97^c^	118^e^

^a^Above ground corresponding to isolates from silk, tassel, ear, and insect.

^b^300 bp fragment, deletion pattern I.

^c^800 bp fragment, deletion pattern II.

^d^Number of isolates belonging to the indicated haplotype.

^e^Number of isolates examined for *omtA* amplicon.

**Table 2:** *omtA* gene polymorphism statistics for *Aspergillus flavus* from a single Mississippi field.

Season/Source/Year	Sequence and haplotype				Nucleotide diversity				Population expansion				
	*N*	*S*	*H*	Hd ± sd	*π*	*K*	*θ*_*s*_	*θ*_*g*_	*D* ^+^	*F* ^+^	*Fs*	*S*	*D*
May–Oct./soil/2006	8	28	4	0.64 ± 0.18	0.020	12.17	0.018	10.79	0.96	0.99	4.86	0.05	0.67
May–Oct./soil/2007	10	26	4	0.53 ± 0.18	0.014	8.77	0.015	9.19	1.11	0.88	5.02	0.03	−0.21
May–Oct./soil/2008	8	39	5	0.78 ± 0.15	0.023	14.25	0.025	15.42	0.15	0.02	3.32	0.16	−0.40
May–Oct./above ground/2006	9	28	5	0.86 ± 0.08	0.022	13.27	0.017	10.30	1.32	1.51	3.92	0.09	1.44
May–Oct./above ground/2008	10	32	5	0.66 ± 0.16	0.011	6.88	0.020	12.01	−2.36^*^	−2.58^*^	2.45	0.23	−2.05^*^
Jan.–April/soil/2006	15	28	5	0.59 ± 0.14	0.015	9.27	0.014	8.80	0.92	0.84	5.34	0.02	0.22
Jan.–April/soil/2007	23	26	4	0.52 ± 0.09	0.016	9.84	0.011	7.04	1.20	1.51	11.4	0.00	1.49
Jan.–April/soil/2008	17	24	2	0.22 ± 0.12	0.008	5.29	0.011	7.09	1.59^*^	0.98	10.3	0.00	−1.02
Jan.–April/soil/2009	5	25	3	0.80 ± 0.16	0.024	14.80	0.020	12.00	1.73^*^	1.86^*^	4.74	0.09	1.73
Jan.–April/plant debris/2006	5	23	2	0.40 ± 0.23	0.015	9.20	0.018	11.04	−1.23^*^	−1.32	6.64	0.02	−1.23
Jan.–April/plant debris/2007	4	23	3	0.83 ± 0.22	0.001	1.00	0.001	1.09	−0.70	−0.60	−0.88	0.95	−0.70
Jan.–April/plant debris/2008	4	23	2	0.66 ± 0.20	0.025	15.33	0.021	12.54	2.28^*^	2.38^*^	6.76	0.03	2.28^*^

Combined	118	68	24	0.61 ± 0.04	0.018	11.08	0.023	13.94	−4.07^*^	−3.13^*^	1.83	0.20	−0.65

Note: *N* = number of sequences; *S* = number of variable sites; *H* = number of haplotypes; Hd = haplotype diversity ± standard deviation; *π* = nucleotide diversity; *K* = mean number of pairwise nucleotide differences; *θ*_*s*_ = theta per site; *θ*_*g*_ = theta per gene; *D* ^+^and *F* ^+^ statistics (Fu and Li 1993) [21]; *Fs*  Fu's  *F*'s statistic; Strobeck *S* statistic (Strobeck 1987) [22]; *D* = Tajima's (1989) [23] statistic; ^*^ *P* < 0.05.

**Table 3:** *omtA* observed *F*_ST_ values (below) and gene flow (*N*_*M*_) calculated with Nei (1982) [24] for *Aspergillus flavus* isolates collected from twelve spatiotemporal locations from a single corn field in Mississippi.

*F*_ST↓_ *N*_*M*→_	1	2	3	4	5	6	7	8	9	10	11	12
1	—	1.72	2.62	11.73	10.95	29.37	0.34	10.88	3.26	4.10	1.68	2.42
2	0.12	—	0.62	0.98	0.93	1.58	1.29	1.35	0.57	0.57	3.00	22.19
3	0.00	0.37^*^	—	4.51	3.78	4.77	0.11	3.11	3.86	4.74	0.58	0.65
4	−0.05	0.29^*^	−0.00	—	87.94	42.29	0.32	15.52	9.36	18.02	1.25	1.57
5	−0.07	0.27^*^	−0.02	−0.08	—	39.32	0.23	12.87	9.04	18.43	1.05	1.30
6	−0.06	0.22^*^	0.04	−0.04	−0.06	—	0.60	22.67	6.48	7.77	2.22	2.99
7	0.54^*^	0.17	0.77^*^	0.65^*^	0.67^*^	0.57^*^	—	0.34	0.15	0.18	0.54	0.70
8	−0.09	0.17	−0.01	−0.06	−0.08	−0.06	0.54^*^	—	5.18	6.05	1.42	1.94
9	0.02	0.40^*^	−0.02	−0.03	−0.05	0.01	0.77^*^	−0.02	—	17.39	0.63	0.74
10	0.03	0.44^*^	0.00	−0.03	−0.05	0.01	0.79^*^	−0.00	−0.05	—	0.73	0.83
11	0.08	−0.01	0.31	0.27^*^	0.24^*^	0.21	0.30	0.12	0.39^*^	0.45^*^	—	2.65
12	0.02	−0.15	0.29	0.19	0.18	0.12	0.25	0.06	0.33^*^	0.39^*^	−0.08	—

1 = growing season soil 2006; 2 = growing season above ground 2006; 3 = overwintering plant debris 2006; 4 = overwintering soil 2006; 5 = growing season soil 2007; 6 = overwintering soil 2007; 7 = overwintering plant debris 2007; 8 = growing season soil 2008; 9 = growing season above ground 2008; 10 = overwintering soil 2008; 11 = overwintering plant debris 2008; 12 = overwintering soil 2009; ^*^significant at *P* < 0.05.
